# Short- and Long-term Results of Glaucoma Valve Implantation for Aniridia-related Glaucoma: A Case Series and Literature Review

**DOI:** 10.4274/tjo.galenos.2019.07348

**Published:** 2019-09-03

**Authors:** Gülizar Soyugelen Demirok, Ümit Ekşioğlu, Mehmet Yakın, Ahmet Kaderli, Sema Tamer Kaderli, Firdevs Örnek

**Affiliations:** 1 Ankara Training and Research Hospital, Ophthalmology Clinic, Ankara, Turkey

**Keywords:** Aniridia-related glaucoma, congenital aniridia, glaucoma, glaucoma drainage devices

## Abstract

**Objectives:**

To report the results obtained from glaucoma drainage device (GDD) implantation in patients with aniridia-related glaucoma and to review the literature.

**Materials and Methods:**

We retrospectively reviewed 6 patients who underwent GDD implantation for glaucoma secondary to congenital aniridia between April 2001 and February 2015. Data on age at surgery, gender, laterality, surgeries before GDD implantation, GDD model, concomitant ocular disorders, visual acuity, and intraocular pressure (IOP) values before and at 1 and 12 months after GDD implantation, medications, follow-up period, findings during last visit, complications, and course of disease were collected.

**Results:**

Mean age at surgery was 16.00±12.31 years (range 5-37 years). Mean IOP was 33.00±12.11 (range 22-50) mmHg just before the GDD implantation with a mean of 3.5±1.2 medications. Mean IOP 1 month after implantation was 16.33±4.22 (range 12-24) mmHg with a mean of 1.5±0.8 medications; at 12 months, mean IOP was 19.50±4.76 (range 15-26) mmHg with 3.0±0.8 medications. At the last follow-up visit, IOP was 21.16±4.07 (range 16-26) mmHg with a mean of 3.33±0.51 medications. Mean follow-up was 19.16±8.8 (range 12-36) months. Surgical success rates were 83.3%, 66.6%, and 50.0% at 1 month, 12 months, and the last visit, respectively.

**Conclusion:**

GDD implantation was effective in controlling aniridic glaucoma with a success rate of 83.3% at 1-month follow-up and 66.6% at 1-year follow-up. Therefore, it should be considered as an initial surgical treatment for aniridic glaucoma; the clinician should be alert for concomitant ocular disorders.

## Introduction

Congenital aniridia is a rare bilateral panocular disorder caused by a mutation in the *PAX6* gene, which is located on chromosome 11p.^1,2^ The disease is characterized by partial or complete hypoplasia of the iris, corneal opacification, cataract, glaucoma, nystagmus, foveal, and optic disc hypoplasia.^3,4,5^ The incidence of glaucoma associated with aniridia ranges from 6% to 75% and remains one of the most challenging features of aniridia.^6^ Although several studies have attempted to clarify the mechanism underlying aniridic glaucoma, the currently accepted one is progressive anterior rotation of the rudimentary iris, leading to angle closure. On the other hand, maldeveloped anterior chamber angle and undeveloped Schlemm canal are other possible mechanisms.^7^

Aniridic glaucoma is frequently refractory to medical treatment, and patients eventually need surgery.^8^ Surgical options include goniotomy, trabeculotomy, trabeculectomy, glaucoma drainage devices (GDD), and cyclodestructive procedures, but there is no consensus as to the best surgical treatment.^7,9,10,11^ Visual prognosis is generally poor due to intractable glaucoma or concomitant ocular disorders.

In this study, we report the results obtained from GDD implantation in patients with congenital aniridia-related glaucoma, along with a literature review.

## Materials and Methods

This retrospective, noncomparative study was approved by the Review Board of Ankara Training and Research Hospital and adhered to the provisions of the Declaration of Helsinki for research involving human subjects.

### Patient Data

Medical records of patients who underwent GDD implantation for glaucoma secondary to congenital aniridia between April 2001 and February 2015 were analyzed. The following data were collected: age at time of surgery, gender, laterality, surgeries before GDD implantation, GDD model, concomitant ocular disorders, visual acuity, intraocular pressure (IOP) values before and at 1 and 12 months after GDD implantation, medications, follow-up period, findings in last visit, complications, and disease course. Surgical success for GDD implantation was defined as postoperative IOP between 5 and 22 mmHg with or without glaucoma medication. IOP was measured by Goldmann or Perkins applanation tonometry.

The diagnosis of limbal stem cell deficiency (LSCD) was made largely on clinical grounds. Loss of limbal anatomy, corneal conjunctivalization, persistent epithelial defects, and irregular fluorescein staining were considered as LSCD.

After establishing the prediagnosis, renal ultrasound and genetic analysis were performed if necessary.

### GDD Implantation Technique

Under peribulbar or general anesthesia, a 8-0 silk suture was inserted into the superior limbal cornea. Conjunctival dissection was performed posteriorly by blunt dissection in the superior-temporal quadrant and a fornix-based opening was created. The GDD implants (Ahmed Glaucoma Valve-AGV) (New World Medical, Inc., Rancho Cucamonga, CA) were irrigated with 2 mL of balanced saline solution (priming). The plate was secured to the superficial sclera 8 mm from the limbus using 2 interrupted 6-0 absorbable sutures after passing through the holes. The tube was cut to extend 1-3 mm beyond the posterior surgical limbus. The anterior chamber was entered 2.0 mm posterior to the limbus with a 22-gauge needle directed parallel to and just anterior to the iris plane, and viscoelastic was administered. The tube was inserted through the needle tract using a smooth forceps, making sure not to touch the iris or cornea. The tube was secured to the sclera using 10-0 nylon sutures, then covered with the donor graft. The conjunctiva was closed using a 10-0 nylon suture. A subconjunctival antibiotic and steroid injection was performed.

## Results

Six eyes of 6 patients (5 males, 1 female) who underwent GDD implantation for glaucoma secondary to aniridia were included in the study. The patients’ demographics and preoperative clinical findings are given in Table 1. The mean age at surgery was 16.00±12.31 (range 5-37) years. Preoperatively, the mean IOP was 33.00±12.11 (range 22–50) mmHg using a mean of 3.5±1.2 anti-glaucomatous medications. In 4 cases (66.6%), lens extraction was performed due to cataract before GDD implantation.

The patients’ postoperative outcomes are shown in Table 2. At 1 month, the mean IOP was 16.33±4.22 (range 12-24) mmHg with a mean of 1.5±0.8 medications, and surgical success was 83.3%. At 12 months, surgical success was 66.6% and mean IOP was 19.50±4.76 (range 15-26) mmHg with a mean of 3.0±0.8 medications.

The average follow-up was 19.16±8.8 (range 12-36) months. Mean IOP was 21.16±4.07 (range 16-26) mmHg with a mean of 3.33±0.51 medications, and surgical success was 50.0% at the last visit.

In Patient 1, GDD implantation was complicated by tube exposure at postoperative 1 month and retinal detachment associated with vitreous hemorrhage. The exposed tube area was repaired with fascia lata, and detachment surgery was performed. No other complications associated with GDD were observed for 6 months, after which the patient continued follow-up in his area of residence.

The right eye of Patient 4 was complicated with vitreous hemorrhage and retinal detachment after trabeculectomy. Therefore, GDD implantation was considered as the first surgical treatment for the left eye.

Patient 5, who underwent penetrating keratoplasty and allogenic keratolimbal allograft from cadaver 3 years before GDD implantation, developed corneal graft rejection and total corneal leucoma. A second keratoplasty was not considered due to risk of graft rejection.

**Figure 1:**
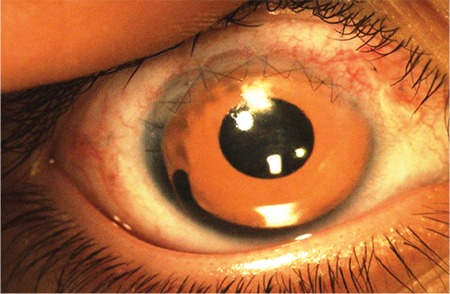
Aniridia intraocular lens (lens with artificial iris)

Patient 6 received an aniridia intraocular lens implantation after lens extraction (Figure 1). This was the only case associated with Wilms’ tumor, bilateral sporadic aniridia, genitourinary abnormalities, and mental retardation syndrome.

## Discussion

Aniridia is a severe panophthalmic disorder characterized by the presence of only a rudimentary iris peripherally. It is often difficult to manage due to progressive features with many ocular complications such as cataract, glaucoma, and corneal opacity.^3^ Fortunately, the incidence of disease is between 1:64,000 and 1:100,000.^12^

Aniridic glaucoma is thought to be due to developmental abnormalities in the drainage angle or progressive closure of the angle by the rudimentary iris. Treatment is very difficult, and a surgical approach is often required due to limited response to medical therapy. The rate of response to medical treatment, including miotic eye drops and oral carbonic anhydrase inhibitors, was reported as 38.7%.^13^ In addition, successful results were not obtained with argon laser trabeculoplasty or diode laser photocoagulation in patient with aniridic glaucoma.^7,14^ Prophylactic goniotomy to separate the abnormal extensions of the rudimentary iris to the angle was reported to be an effective method for preventing or delaying the onset of aniridic glaucoma.^10^ However, glaucoma does not develop in a significant proportion of those with aniridia, so it should not be used routinely.

Ciliary body destruction was performed with cyclocryotherapy in patients with aniridic glaucoma by Wagle et al.^15^ However, it was not presented as a first-line treatment because of serious complications such as hypotony, phthisis bulbi, cataract, and vision loss. Considering that most cases occur in young patients and affect both eyes, these complications could be more destructive. Wiggins and Tomey^7^ reported a success rate of only 25% after cyclocryotherapy in aniridic glaucoma. Conversely, Kirwan et al.^16^ found cyclodestruction with cyclophotocoagulation diode laser as an effective treatment in refractory pediatric glaucoma patients, including 5 aniridic eyes.

Many studies have tried trabeculectomy in aniridic glaucoma. While Grant and Walton^13^ reported failures in all eyes that underwent trabeculectomy, Nelson et al.^6^ reported successful outcomes in 11 of 14 eyes even 1 year after trabeculectomy. Okada et al.^9^ reported successful IOP control in their study, including 10 eyes of 6 patients with 14.6-month follow-up.^9^ Adachi et al.^17^ achieved IOP control for 1 year in only 1 of 5 patients who underwent trabeculectomy as an initial treatment. The fibrotic nature emerging from cell adhesion of aniridia may be the main reason for higher risk of treatment failure than that seen in patients with primary glaucoma who undergo the same treatment. This situation requires the use of antimetabolites (e.g., 5-fluorouracil, mitomycin C), which damage stem cells and accelerate the progression of corneal complications.

In the present study, GDD implantation was performed as an initial procedure for aniridic glaucoma in 6 eyes of 6 patients. While our success rate was 83.3% at 1 month, it fell to 66.6% at 12 months, and was 50.0% at the last follow-up visit. This decrease in success rate over time is consistent with the literature. Arroyave et al.^11^ presented 8 eyes of 5 patients who underwent GDD placement as initial surgery (6 eyes) or after unsuccessful glaucoma surgeries (2 eyes). The rate of success, which was defined as postoperative IOP of 21 mmHg or less with or without glaucoma medications, was 100% at 6 months and 88% at 1 year. Hence, the success rate decreased over time similarly to our result. This may be attributed to the fibrotic process of this disease, which leads to reduced drainage over time. The latest study on GDD implantation for aniridia-related glaucoma was presented in 2014 by Almousa and Lake,^18^ who reported successful IOP control in 87% of eyes (7 of 8 eyes). In addition, the success rate varied between 66% and 100% in another 5 studies in which Molteno GDD was used for aniridic glaucoma.^7,17,19,20,21^ Previously published data are shown in Table 3. The success rate of GDD surgery in studies, including ours, seems good despite the poor prognosis of the disease.

As observed in a series which included the medical records of 128 eyes of 64 patients with aniridic glaucoma, one-fourth of patients had phthisis in at least 1 eye at the last postoperative follow-up.^22^ Okamoto et al.^23^ suggested that ciliary body hypoplasia could be responsible for the higher rates of phthisis compared to other pediatric glaucomas in these patients. However, there were no phthisis or hypotony in our series; this may be due to the small number of patients, or selecting the GDD implantation as an initial surgery for glaucoma.

### Study Limitations

The small number of cases, the nonadherence to follow-up of some patients, and the retrospective, noncomparative design should be considered limitations of this study. Even so, this is the second largest case series in the literature.

## Conclusion

In conclusion, GDD implantation for aniridia-related glaucoma achieved successful IOP control in most patients. Hence, GDD placement could be considered as an initial surgical treatment when IOP remains uncontrolled, despite maximal medical therapy in aniridic glaucoma. Randomized studies are needed to determine the best surgical method for aniridia-related glaucoma.

## Figures and Tables

**Table 1:**
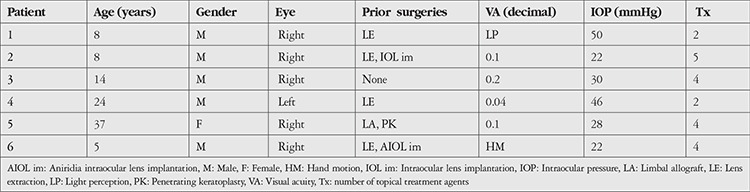
Patients’ demographics and preoperative clinical findings

**Table 2:**
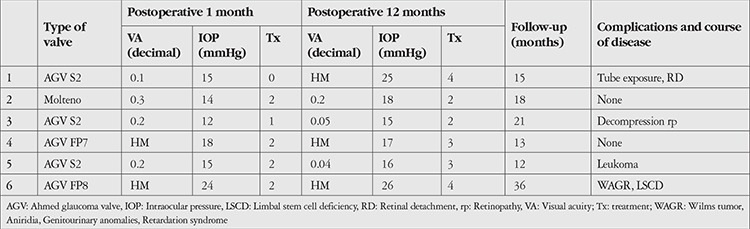
Patients’ surgical outcomes

**Table 3:**
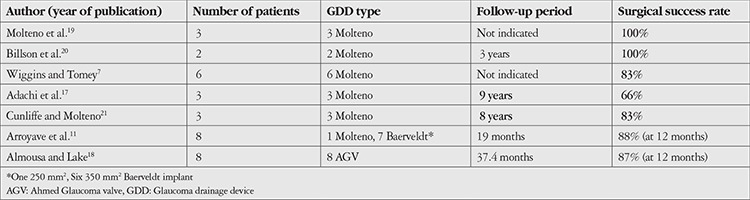
Previously published data on glaucoma drainage device in eyes with aniridic glaucoma
